# Case report: a 37-year-old male with telangiectasias, polycythemia vera, perinephric fluid collections, and intrapulmonary shunting

**DOI:** 10.1186/2052-1839-14-11

**Published:** 2014-07-22

**Authors:** Javed Khan, David B Sykes

**Affiliations:** Room 91 Old Doctors Hostel, Khyber Teaching Hospital Peshawar, Peshawar, 25000 Pakistan; Division of Hematology, Massachusetts General Hospital Cancer Center, 55 Fruit Street, Boston, MA 02114 USA

**Keywords:** Erythrocytosis, Erythropoietin, Monoclonal gammopathy, Perinephric fluid, Polycythemia, TEMPI

## Abstract

**Background:**

The TEMPI syndrome was recently described in 2011, and is characterized by the constellation of five hallmarks: *T*elangiectasias, *E*rythrocytosis and elevated Erythropoietin, *M*onoclonal gammopathy, *P*erinephric fluids collections, and *I*ntrapulmonary shunting. The underlying pathophysiology is unknown, though it has been postulated that the monoclonal gammopathy may play a causal role.

**Case presentation:**

A 37-year-old non-smoking male presented to our institution with a fever and the sensation of fullness in the right flank. His exam was notable for telangiectasias, clubbing of the fingernails, plethora, and a palpable bulge in the right flank. Renal ultrasound demonstrated bilateral perinephric fluid collections. Laboratory evaluation revealed erythrocytosis with low serum erythropoietin, and testing for the JAK2V617F mutation was positive, confirming a diagnosis of polycythemia vera. Though his room air saturation was normal at rest, it decreased dramatically with exercise, felt to be secondary to microscopic intrapulmonary shunting.

The patient’s presentation is very similar to that of the TEMPI syndrome, a very rare syndrome of which there have been six published cases. In contrast to the TEMPI syndrome where the erythrocytosis is driven by highly elevated serum erythropoietin, our patient was found to have polycythemia vera. Also in contrast to the other patients with TEMPI syndrome, our patient did not have an identifiable monoclonal gammopathy.

Our patient responded to treatment with hydroxyurea. His erythrocytosis, perinephric fluid collections, and telangiectasias resolved over the course of six months. The intrapulmonary shunting has continued to gradually improve with treatment, suggesting that this is an entirely reversible process.

**Conclusion:**

Our case is the first to describe the combination of polycythemia vera, telangiectasias, perinephric fluid collections, and intrapulmonary shunting. The presentation is highly similar to the previously described TEMPI syndrome, though calls into question the potential importance of the monoclonal gammopathy. Our patient demonstrated a response to treatment with hydroxyurea, while patients with the TEMPI syndrome have shown responses to plasma-cell directed therapies such as bortezomib.

## Background

The description of the TEMPI syndrome, and a small case series of three patients, was recently published by Sykes et al. in the New England Journal of Medicine [1]. The syndrome was designated TEMPI by the authors based on five key characteristics:

-  
*T*elangiectasias
-  
*E*rythrocytosis & elevated serum Erythropoietin
-  
*M*onoclonal gammopathy
-  
*P*erinephric fluid collections
-  
*I*ntrapulmonary shunting

Further review of the literature identified three additional patients [2–4], but failed to reveal a pathophysiological understanding of this syndrome. Two additional patients have also been identified, but not published, bringing the total to eight known living patients with the TEMPI syndrome (Sykes DB, personal communication).

## Case presentation

**Figure 1:**
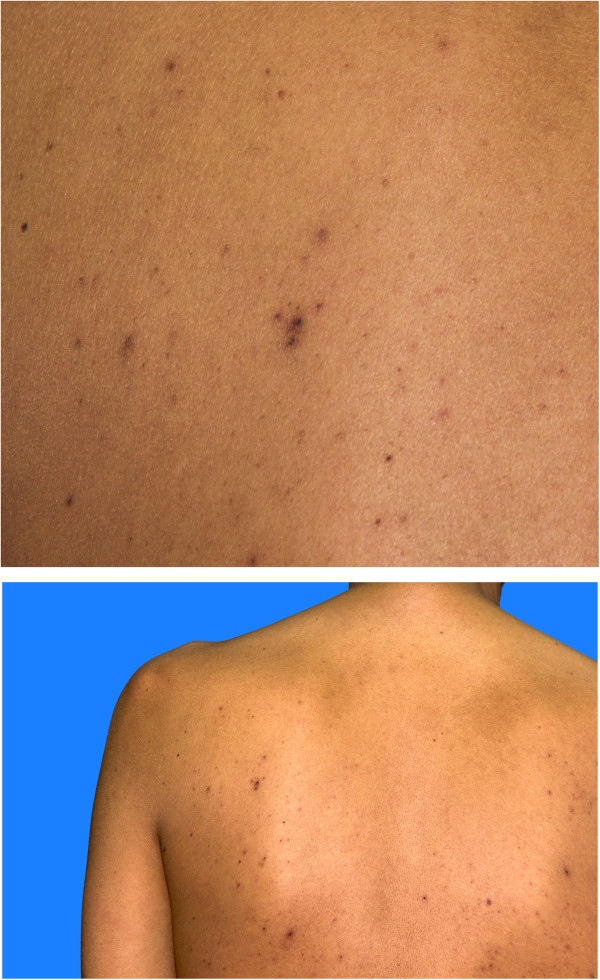
**Telangiectasias were noted on the back and trunk.**

A 37 year-old male non-smoking farmer with no prior medical history initially presented to his physician with concerns of flank pain and fever. On interview, he had noted telangiectasias (he described them as ‘small boils’) for approximately one year prior to presentation. He was febrile to a temperature of 102°F. He had normal room air oxygen saturation at rest (98%). His physical exam was notable for plethora, clubbing in the nails of the hands, telangiectasias on the back (Figure 1) and a palpable mass in the right abdomen. Testing of the stool for occult blood was negative.

**Figure 2:**
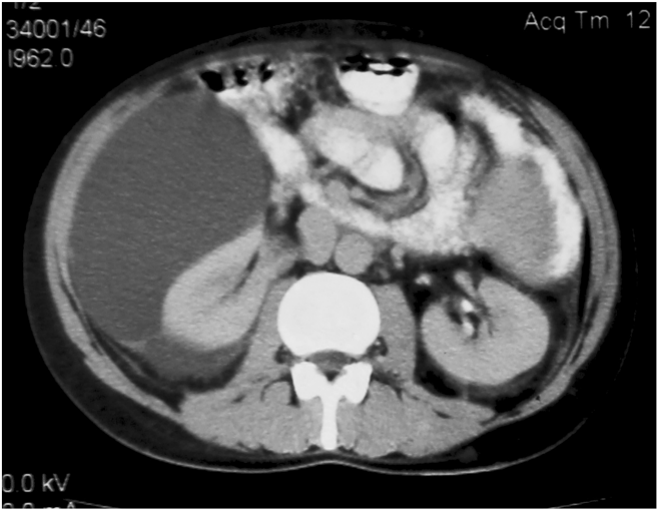
**A contrast enhanced CT scan of the abdomen demonstrates showing a large right-sided perinephric fluid collection.**

An abdominal ultrasound revealed perinephric fluid collections, larger on the right side than on the left side. A computed tomography scan of the abdomen confirmed the ultrasound findings and demonstrated a right-sided perinephric fluid collection measuring 10 cm × 12 cm × 17 cm as well as a very small collection on the left side (Figure 2) and mild splenomegaly. Of particular note was the lack of renal cysts and the lack of any intraparenchymal fluid collections.

The patient underwent an extensive laboratory evaluation (Table 1). His complete blood count demonstrated polycythemia and a neutrophil-predominant leukocytosis. The serum electrolytes were normal; BUN and creatinine were slightly elevated. The evaluation of his erythrocytosis revealed a suppressed serum erythropoietin of 2 mIU/ml (normal range 4–17 mIU/ml) and a heterozygous JAK2V617F mutation consistent with a diagnosis of polycythemia vera. Our patient did not have a history of thrombosis and did not describe any symptoms consistent with erythromelalgia.

**Table 1:** **Laboratory evaluation**

Parameter	Result	Normal range
WBC (TLC)	12,000	4000-11000/cmm
RBC	9.94	4.4-5.5 million/cmm
Hb	19.6	14-18 gm/dl
HCT (PCV)	66.7	40-45%
MCV	67	80-96 fL
MCH	19.7	27-32 pgm
MCHC	29.4	30-35/dl
Platelets	572,000	150,000—400,000/cmm
Differential (DLC)		
Neutrophils	90%	
Lymphocytes	08%	
Monocytes	02%	
Eosinophils	00%	
RBC MORPHOLOGY		
Anisocytosis	+ive	
Hyphochromia	+ive	
Microcytosis	+ive	
Sodium	133	135-145 mmol/L
Potassium	4.7	3.4-4.8 mmol/L
Chloride	105	100-108 mmol/L
Blood Urea Nitrogen	37	15-40 mg/dl
Creatinine	1.9	0.9-1.3 mg/dl
Glucose	100	70-110 mg/dl
Total Proteins	5.7	6-7 g/dl
Albumin	3.5	3.2-5 g/dl
Globulin	2.2	1.8-3.6 g/dl
Bilirubin (total)	1.2	0.0-1.0 mg/dl
ALT (SGPT)	17	10-55 U/L
AST (SGOT)	14	10-40 U/L
Uric acid	9	3.4-7.2 mg/dl
PT	14	(Control = 12 sec)
aPTT	34	(Control = 32 sec)
HBsAg	Non-Reactive	Non-Reactive
Anti HCV Abs	Non-Reactive	Non-Reactive
HIV	Non-Reactive	Non-Reactive

His red blood cells were initially microcytic (MCV 67 fl), though this corrected into the normal range with oral iron supplementation. A hemoglobin electrophoresis was not performed.

The serum protein electrophoresis was normal, and immunofixation did not reveal any monoclonal bands. Evaluation of the urine did not reveal any Bence-Jones protein. The serum uric acid was slightly elevated, this was felt to be due to increased RBC turnover in the setting of polycythemia.

The patient’s urinalysis was normal. He underwent aspiration of the right-sided perinephric fluid collection and a drainage catheter was left in place. Examination of the fluid revealed a clear fluid with few RBC and few inflammatory cells. Cytology revealed no malignant cells. The fluid had very low levels of protein, glucose, and triglycerides. The fluid amylase was 15 U/L. The fluid creatinine was 0.74 mg/dL, arguing against the possibility of a urinoma.

He next underwent evaluation of his exercise tolerance and oxygen saturation. His room air oxygen saturation was 95% at baseline but decreased to 75% after 2 minutes of exercise on the treadmill, at which point the test was stopped. The patient’s ECG and echocardiogram were normal. Microscopic intrapulmonary shunting was suspected.

The abdominal CT scan and voiding urogram confirmed the large right-sided perinephric fluid collection. The collection compressed the right kidney though the excretion of dye was normal. The left kidney showed only a small amount of perinephric fluid. The CT urography showed no connection of the fluid collection with renal collecting system.

Cultures of urine, blood, and perinephric fluid showed no aerobic or anaerobic growth. Examination of thick and thin blood smears showed no malarial parasites.

Testing for Anti-nuclear antibodies (ANA), Anti-Smooth Cell Antibodies (ASMA), and Anti-mitochondrial Antibodies (AMA) was negative.

The patient was seen in consultation by radiology, nephrology, surgery, and medical teams. He underwent therapeutic phlebotomy to remove one unit of blood. He then initiated therapy with hydroxyurea (500 mg given twice daily by mouth). Over the first two weeks of treatment, daily drainage output decreased from 1500 ml daily to 50 ml daily, and the drain was removed.

The patient was discharged. On his first follow-up visit (approximately three months) his hemoglobin was 17.5 mg/dl and an ultrasound confirmed that his perinephric fluid collections had resolved. On his second follow-up visit (approximately six months) his hemoglobin was 16.4 mg/dl. His telangiectasias had also almost completely resolved and he felt that his exercise tolerance was improved though formal exercise pulse oximetry was not performed.

## Conclusions

Our patient presented with four of the five hallmark characteristics of the TEMPI syndrome, including telangiectasias, erythrocytosis, perinephric fluid collections, and intrapulmonary shunting. Of particular note, our patient lacked the monoclonal gammopathy common to patients with TEMPI syndrome. To date, 8 living patients have been identified with the TEMPI syndrome, all possess a monoclonal gammopathy (Sykes DB, personal communication). Furthermore, in contrast to these other eight patients, our patient’s erythrocytosis was due to polycythemia vera rather than to an elevated serum erythropoietin level.

Our patient appears to have a unique constellation of symptoms, not previously described in the literature. The pathophysiology underlying the telangiectasia formation and intrapulmonary shunting is not clear. In patients with the TEMPI syndrome, the authors postulated that the monoclonal gammopathy might be playing a causal role [5]. However, our patient presented with both telangiectasias and intrapulmonary shunting in the absence of a monoclonal gammopathy, arguing against this hypothesis.

The patients with TEMPI syndrome also have a characteristically elevated serum erythropoietin level, elevated to 10–500 times the upper limit of normal. However, our patient had a low serum erythropoietin. This also argues against the possibility that EPO is acting as a proliferative signal in the abnormal blood vessel formation (telangiectasia and microscopic intrapulmonary shunting) that is characteristic of patients with the TEMPI syndrome.

In patients with the TEMPI syndrome, partial or complete responses have been seen upon treatment with the proteasome inhibitor bortezomib (Kwok 2012 [3] and Schroyens 2012 [5]). Our patient has had a response, in terms of his erythrocytosis, telangiectasias, and perinephric fluid collections, to simple treatment with hydroxyurea. We have not yet seen a complete response in terms of his intrapulmonary shunting as manifested by his decreased exercise oxygen saturation, though this may simply take longer to reverse.

International collaboration and modern medical search engines have made it possible to identify collections of ultra-rare patients such as ours. We hope that the continued description of these patients will eventually lead to an understanding of their disease pathophysiology and provide a rational means of disease treatment.

## Consent

Written informed consent was obtained from the patient for publication of this case report and for the accompanying images. A copy of the written consent is available for review.

## Abbreviations

TEMPI: Telangiectasias, Erythrocytosis and elevated Erythropoietin, Monoclonal gammopathy, Perinephric fluids collections, and Intrapulmonary shunting.
